# Addressing the Gap: Real-World Evidence of Technology-Enabled Coaching Services for Mental Health

**DOI:** 10.1007/s10488-025-01473-8

**Published:** 2025-09-27

**Authors:** Sara Sagui Henson, Komal Kumar, Kristen M. Van Swearingen, Jessica Watrous, Neha Chaudhary

**Affiliations:** 1 Clinical Strategy and Research Team, Modern Health, San Francisco, CA USA; 2 Health Psychology Doctoral Program, University of North Carolina at Charlotte, Charlotte, NC USA

**Keywords:** Mental health, Digital health, Coaching, Anxiety, Depression, Stress

## Abstract

A quarter of US adults face mental health challenges, yet less than half receive treatment, partly due to therapist shortages. Scaling trained paraprofessionals to provide effective care could address this gap. We evaluated the impact of evidence-based coaching within a real-world blended care digital mental health platform. 266 working adults (62% women, *M*_*age*_=33 years, 40% people of color) newly enrolled (2021–2022) in the platform (Modern Health) completed surveys at baseline and 3-months. Participants primarily engaged with certified professional coaches and could also access therapy and digital resources. We examined pre-post changes in outcomes and differences by baseline clinical status. Participants used an average of 2.5 coaching sessions and improved in distress tolerance (+ 4.1%), perceived stress (-8.4%), self-compassion (+ 5.9%), and mindfulness (+ 2.6%) (*p*s < 0.01). Although elevated risk users (vs. lower risk) reported descriptively greater improvements in distress tolerance (+ 4.7% vs. +3.7%), perceived stress (-10% vs. -3.7%), self-compassion (+ 7.6% vs. +5.2%), and mindfulness (+ 3.3% vs. +2.1%), these differences were not statistically significant (*p*s > 0.05). Depression (-22.5%) and anxiety (-12%) improved (*p*s < 0.001), with elevated risk users reporting greater reductions (*b*=-3.15, *p* < .001 for depression; *b*=-2.61, *p* < .001 for anxiety). Among elevated risk users, 71.7% improved or recovered from depression or anxiety and 96.1% of lower risk users maintained lower depression and anxiety. Technology-enabled coaching, as part of a blended care platform, improved depression, anxiety, and transdiagnostic emotional processes. Participants with higher baseline depression and anxiety reported greater improvements, highlighting coaching’s effectiveness for people with elevated needs. By redefining care and leveraging technology, coaching may be a scalable treatment solution.

**Supplementary Information:**

The online version contains supplementary material available at 10.1007/s10488-025-01473-8.

## Introduction

In 2021, almost a quarter of United States (US) adults faced mental health challenges, yet less than half received treatment (National Institute of Mental Health,^2024^). While a number of complex factors contribute to this treatment gap, such as affordability, accessibility, and stigma (Substance Abuse and Mental Health Services Administration, 2018), therapist shortages present a significant challenge to treatment accessibility. The current healthcare system’s dependence on extensively trained mental health professionals, who invest years in academic and clinical training, inherently creates barriers to widespread service delivery. In the US, almost one in five counties (18%) have reported a shortage of nonprescribing mental health providers, such as licensed psychologists, therapists, and counselors, with rural and low-income populations showing the highest levels of unmet needs (Butryn et al., 2017). This accounts for 47% of the US population, with certain states needing hundreds of new practitioners to address this deficit (American Counseling Association, 2023; Health Resources and Services Administration, 2024).

To improve this gap, strategic innovation is needed to rethink care provision, including who can provide care, to whom, and by what means. One promising solution is to train alternative providers to deliver effective care to a subset of the population based on clinical needs and use technology to scale up access to this care (Naslund et al., 2019). Although people with more severe clinical needs are still best treated by licensed mental health clinicians (e.g., therapists, psychiatrists), paraprofessionals have been leveraged worldwide to address the global mental health treatment gap over the last decade (Anvari et al., 2023; Fernando et al., 2021; Myers et al., 2024).

Paraprofessionals include a diverse group of experts, such as allied health professionals, coaches, lay health workers, peers, religious professionals, and more (Anvari et al., 2023; National Governors Association, 2024; Passmore et al., 2024). Typically, behavioral health paraprofessionals deliver interventions that draw from the principles of psychotherapy (e.g., Motivational Interviewing), to people with lower clinical needs (Abu Dabrh et al., 2025; Clark et al., 2014; Fernando et al., 2021; Myers et al., 2024). Examples range from the Friendship Bench with trained lay health workers in Zimbabwe (Fernando et al., 2021) and African immigrant pastors in the US assisting with mental health care provision in their community (Myers et al., 2024), to paraprofessionals delivering Cognitive Behavioral Therapy with comparable effectiveness to licensed therapists (Montgomery et al., 2010). The World Health Organization even developed the Mental Health Gap Action Programme Intervention Guide in 2017 to assist non-specialized health care workers in providing evidence-based practices (Wainberg et al., 2017).

One type of paraprofessional of particular interest in the US is coaches. There are many definitions of a coach depending on the context (e.g., health coach, wellness coach, career coach). A coach for mental health can be defined as a trained professional who delivers a structured, goal-oriented intervention and uses evidence-based therapeutic and behavioral change approaches to support their client in achieving meaningful change to their thoughts, behaviors, and well-being (Passmore et al., 2024). Scaling coaching services is a potential approach to address the mental health gap (Clark et al., 2014), but the variability in training and technique within the field of coaching leads to common criticisms of the field overall that create challenges for integrating it more broadly into mental and behavioral health care.

First, there is not currently a central credentialing organization for coaches to promote the provision of high-quality mental health support services. Unlike licensed mental health professionals, who must meet rigorous, standardized state-based licensure requirements, coaches are not universally subject to such regulations in order to practice. However, there are several credentialing bodies that have emerged in the past couple of decades to formalize the definition of high-quality, safe, and effective coaching. A few of the leading organizations include the International Coaching Federation (ICF), the National Board of Health and Wellness Coaching, and the Mayo Clinic Wellness Coach Training Program, each with their own rigorous training standards (International Coaching Federation,  2025; Mayo Clinic, 2025; National Board of Health and Wellbeing Coaching, 2024). For example, ICF requires that people complete 60 h of coaching education, 100 h of coaching experience, 10 h of mentor coaching, and pass an exam to be certified as a coach by them (ICF Education and Training Requirements, 2025). The emergence of credentialing organizations (and the employment of certified coaches) represents an important step toward bringing greater legitimacy and standardized quality to the coaching field.

Second, coaching is often perceived as a service designed for mentally healthy individuals seeking to enhance personal (e.g., wellness, relationships) or professional (e.g., career) aspects of their lives. And indeed, coaching can support people with less clinical need in these areas by targeting transdiagnostic emotional processes, which are cognitive and emotional mechanisms believed to underlie multiple mental health conditions (Krueger & Eaton, 2015). There is evidence that coaching improves factors such as perceived stress and coping skills (Clark et al., 2014; Theeboom et al., 2014) and impacts quality of life outcomes like improved life satisfaction (Jeannotte et al., 2021) and enhanced well-being (Prescott et al., 2022). Yet, from a population health perspective (Purtle et al., 2020), scalable interventions must support both prevention for individuals at low risk (helping them build skills and resilience to prevent escalation of symptoms) and targeted support for those with elevated clinical need.

Certified coaches, particularly those trained through the organizations mentioned above, receive extensive education in evidence-based approaches including Motivational Interviewing, Solution-Focused Therapy, Mindfulness-Based Strategies, and Cognitive Behavioral techniques (Clark et al., 2014; International Coaching Federation, 2025; Prescott et al., 2022). This training equips them to effectively support clients with a range of mental health needs. Indeed, research shows that working with a trained coach can lead to significant reductions in depression and anxiety symptoms, with outcomes comparable to those seen in psychotherapy delivered by licensed professionals, and among individuals from diverse racial and ethnic backgrounds (Roos et al., 2024; Sagui Henson et al., 2024; Wu et al., 2021). Because coaches can both enhance emotion regulation skills to prevent symptom escalation and help reduce existing mental health symptoms, coaching may play a valuable role in advancing population-level mental health outcomes.

Third, although coaching is a promising solution, connecting people in need with high-quality coaches is a challenge. Unlike traditional mental health providers–who are most often accessed through healthcare systems and insurance networks–coaches can be found in a variety of ways outside these channels, such as through private practices, employer programs, or digital platforms. This lack of standardization in qualifications and training as well as integration with established referral and insurance systems can make it more difficult for individuals to identify and access qualified coaches. More broadly, limited accessibility to care remains a significant contributor to the mental health treatment gap (Levesque et al., lud^2013^), with barriers including geographic constraints, a shortage of local providers, transportation challenges, and inflexible scheduling.

Digital mental health tools, including telehealth services, that use technology to facilitate care, can uniquely transform access to coaching by mitigating many of these barriers (Aref-Adib & Hassiotis, 2021). Research suggests that a virtual telehealth space can foster strong therapeutic connections and even greater psychological safety (Stubbings et al., 2015). Satisfaction and alliance ratings in videoconferencing settings are comparable to in-person care for both therapy (Simpson & Reid, 2014), and coaching (Sagui-Henson et al., 2022). Technology also enables the development of integrated care delivery systems in which a single digital platform can vet coaches across multiple states, track provider availability, and facilitate coordination between coaching and other services such as therapy or digital self-guided tools. This can help to streamline care pathways and improve continuity of care (Torous et al., 2020).

In addition to increasing access, digital platforms can offer a blended care model, where coaching is integrated with other services such as therapy and self-guided digital tools. This model supports flexibility and autonomy, allowing individuals to personalize their care and combine services to create a more comprehensive and continuous support system. Importantly, when these services are available in one centralized platform (rather than delivered in silos) other modalities such as therapy and self-guided resources can complement one another, such as reinforcing the skills learned in coaching.

A digital coaching service that combines standardized credentialing and technology-enabled access may offer a helpful approach to improving population mental health. For instance, by embedding certified coaches trained to deliver evidence-based strategies into systems that support both prevention for lower-risk individuals and intervention for those with moderate clinical needs, such platforms can address a wide spectrum of mental health concerns. When paired with technology that ensures rapid access to services and enables blended care models, coaching may be a flexible, scalable component of comprehensive mental healthcare. Rigorous evaluation is essential to understanding the impact of such approaches, but technology-enabled coaching solutions represent a promising pathway to expanding access, improving outcomes, and reducing mental health disparities at scale.

To further evaluate the potential of these technology-enabled solutions, we examined the effectiveness of evidence-based coaching services delivered through an employer-sponsored digital mental health platform. The platform uses technology to enhance several aspects of care delivery, including onboarding assessments, care recommendations, provider matching, and telehealth visits. A stratified, blended care model is used where people are recommended a care modality to begin with and then have the flexibility to engage with coaching, therapy, and self-guided digital resources within one central app or website. In this real-world evidence study, participants utilized coaching services with ICF-certified coaches as part of their routine employer-sponsored benefits and surveys were administered before and three months after engaging on the platform.

Our first aim was to evaluate longitudinal changes in the following transdiagnostic emotional processes among people who used coaching as their primary care modality: distress tolerance, perceived stress, self-compassion, and mindfulness. Distress tolerance and perceived stress are risk factors thought to underlie a range of internalizing disorders (Brown et al., 2022; Fassett-Carman et al., 2020), while self-compassion and mindfulness are protective factors that address critical transdiagnostic mechanisms like self-criticism and emotion regulation (Cuppage et al., 2018; Guendelman et al., 2017). Our second aim was to examine changes in depressive and anxiety symptoms, as prior research suggests that targeting both specific symptoms and broader emotion regulation processes can have the greatest impact on mental health outcomes (Sloan et al., 2017). Given the population health framework of this employer-sponsored benefit (i.e., it was designed to serve individuals with a range of mental health needs), we also analyzed outcomes by baseline clinical risk level to reflect the different care goals: symptom reduction for participants with elevated symptoms and symptom maintenance for those with lower symptom burden.

## Methods

### Study Design

This study is part of a larger prospective, longitudinal, observational real-world study of individuals who received services through an employer-sponsored digital mental health benefits platform (Modern Health Inc., San Francisco, CA). The study timeframe was 9/20/21 through 5/31/22. Eligible participants were 18 years or older, based in the US, had enrolled in the digital care platform through their employer, and had access to the platform through a smartphone, tablet, or computer. Data for the current study were collected at baseline before participants engaged with services, and three months following their initiation of services. All participants provided informed consent to participate. This study, and its associated materials, were reviewed and approved by Western Clinical Group (WCG) Institutional Review Board.

### Procedures

The digital mental health platform uses a stratified, blended care model to recommend participants to an initial care pathway based on their clinical need and preferences. Upon registering, participants completed a proprietary onboarding assessment designed to inform this care recommendation. The assessment included validated clinical measures such as the PHQ-9 (for depressive symptoms), GAD-7 (for anxiety symptoms), and WHO-5 (for well-being), along with questions about participants’ topic of focus, level of functional impairment, and care modality preference (e.g., “on my own,” “with a small group,” “one-on-one,” or “I’m not sure”).

A proprietary triage algorithm, developed by clinical experts and software engineers, synthesized this onboarding data to generate a personalized care recommendation. This recommendation considered clinical severity, functional impairment, care preferences, and presenting concerns to help match each participant with an appropriate starting point for care. Participants with higher symptom severity or more acute concerns were typically recommended therapy or coaching, while those with lower symptom burden were more often directed to digital resources. Users retain full autonomy and may choose to engage in any combination of services regardless of their recommendation.

Participants received their care recommendation through a screen in their member portal (accessible via web or mobile), which displayed their topic of focus, recommended care type (e.g., therapy, coaching, digital resources, group sessions), and a rationale for the recommendation. They could then choose to schedule care immediately or explore other available services. Care recommendations presented the suggested type of service as a starting point and did not include prescribed amounts, content, or sequences of care. However, members did receive prompts through email and the app to engage in certain activities, like completing assessments, using their allotted therapy and/or coaching sessions, and engaging in digital content. Regardless of recommendation, participants retained full access to all available services on the platform.

All participants in this study engaged with the platform naturalistically as part of routine care and were additionally invited to complete a series of surveys to measure the effectiveness of this care. After onboarding, participants who met eligibility criteria for this survey study were invited via email to complete a screening questionnaire that captured additional demographic details. Enrollment quotas based on race/ethnicity (per US Census proportions), gender, age, and mental health symptom severity were used to ensure a demographically balanced and representative sample. Once a quota for a demographic group was filled, adjustments to the screening form prevented further enrollment from that category.

Participants who met enrollment criteria proceeded to a consent form. Those who consented received a baseline survey via email, followed by two reminders at two and four days. They had up to one week after the final reminder to complete the survey, ensuring baseline data reflected the beginning of platform use. A follow-up survey was sent three months later. Between the baseline and 3-month follow-up survey, participants engaged naturalistically with the platform. Although the PHQ-9 and GAD-7 were collected during routine onboarding, all data analyzed in this study, including those clinical assessments and all the measures of transdiagnostic emotional processes, were collected specifically at baseline and 3-month follow-up for the purposes of this prospective survey study. Each survey required 30–45 min to complete, and participants received a $25 digital gift card for each completed survey. Surveys were administered using the Qualtrics online platform.

### Digital Mental Health Services

#### Coaching

Participants had access to 30-minute, one-on-one remote coaching sessions, which were led by certified coaches via a videoconferencing platform (Zoom). All coaches were non-licensed providers with an International Coaching Federation (ICF) certification which is recognized as a leading credentialing program for coach practitioners globally. The requirements for this certification included attending 60 h of coaching education, completing 100 h of coaching experience, and 10 h of mentor coaching, as well as receiving a passing score on the Associated Certified Coach Exam (International Coaching Federation, 2025). This certification ensured a minimum standardization for delivering high-quality care.

Coaches applied through the platform’s provider application page, where they submitted information and underwent a structured application and screening process. Coaches were screened by the company’s internal provider network team to ensure they were ICF certified, used evidence-based practices (drawn from Cognitive Behavioral techniques, Acceptance and Commitment techniques, Motivational Interviewing), and upheld cultural humility and ethics. Screening included reviewing responses to sample case studies, written assignments, and a sample coaching session. The vetting focused on ensuring coaches were well-qualified and could deliver culturally centered, effective care.

The coaches included in this study were not a predefined or exclusive subset but rather drawn from the full pool of credentialed coaches available in the platform’s network who provided routine care to participants during the enrollment period. Before providing services, coaches completed at least 6 h of platform-specific training, which covered evidence-based practices, culturally centered care, identifying high-risk situations that may require a therapist or crisis resource referral, and the company’s proprietary blended care model. Coaches continued to receive on-demand and live training, case consultations with licensed clinicians, and quality monitoring (e.g., user feedback, therapeutic alliance ratings). Training was delivered by the company’s clinical strategy and experience team consisting of coaching specialists, licensed psychologists, social workers, and board-certified psychiatrists.

The quality management process was guided by the six aims of healthcare quality (Institute of Medicine, 2001) and overseen by a Quality Improvement Advisory Committee (a team of legal, compliance, provider operations, and clinical experts) to ensure that care that is safe, timely, effective, efficient, equitable, and patient-centered. Metrics monitored included member symptom improvement, therapeutic alliance ratings, qualitative feedback, and provider consultation and training. Continuous improvement efforts were informed by a Global Inclusion Council and clinical team. Incident management was supported through safety review protocols to review incidents and implement corrective actions. Satisfaction and therapeutic alliance with coaches on this platform have been high (e.g., 4.7 + out of 5) in previous studies (Maistrello et al., 2024; Sagui Henson et al., 2024; Sagui-Henson et al., 2022a).

Participants could access coaching through a care recommendation or by self-referral. A proprietary matching algorithm matched participants to a list of coaches based on topic of focus, language (55 + languages at the time of this study), availability, and other personal preferences (e.g., coach gender or race/ethnicity). Participants could schedule directly through the app or web portal. The platform’s provider network and real-time tracking ensure rapid access to care; the list of providers had session availability within a few days, with at least one provider having availability within 24 h. The matching algorithm is a core component of the platform, designed to surface providers who are most relevant to a participant’s clinical needs and preferences. It also considers coach factors like language, credentials, time zone, and availability, which are critical to effective care delivery. Participants could also filter by coach race/ethnicity, and look for coach gender, specialization, or time slots to further refine the list of providers and personalize their provider search.

The provider network coverage system leveraged a global network of coaches and was designed to ensure that the platform could offer the quickest access to high-quality providers, with an average time to the first available session being around one day. This system is supported by the platform’s proprietary technology, which monitors real-time provider availability, capacity tracking, and proactive alerts for providers globally. There was no minimum number of sessions recommended to participants. There was a cap on the maximum number of sessions available for the year and participants were informed of that when they registered. The platform did not prescribe a specific number or cadence of sessions. Instead, engagement was determined by the participant and their coach.

Coaches were not required to follow any single therapeutic protocol during a visit. They followed best practices and policies and procedures outlined by the company and were encouraged to leverage evidence-based techniques as they deemed appropriate to meet the unique needs of everyone. Coaches identified goals the participant wanted to work towards, used evidence-based principles like Cognitive Behavioral techniques or Acceptance and Commitment techniques to help the participant think about self-beliefs or behaviors impeding on those goals, and explored action plans for the participant to work towards desired outcomes. Coaches and participants were also able to communicate by messaging within the app or website. Coaches were not informed whether the individuals they were working with were study participants. This helped to preserve the naturalistic quality of care and minimize bias in how services were delivered.

#### Therapy, Self-Guided Digital Resources, and Group Psychoeducation Sessions

Participants used coaching as their primary form of care, yet within the blended care model, they also had access to therapy, self-guided digital resources, and group psychoeducation sessions to supplement their coaching care.

Participants also had access to 50-minute, one-on-one remote therapy services with licensed therapists with an advanced degree in clinical psychology or a related field (e.g., PhD, PsyD, LCSW, LMFT, or LPC) and skilled in evidence-based practices. Participants with more severe clinical need at baseline were recommended to begin care with therapy. Like coaching, therapy was delivered via a videoconferencing platform and the number of therapy sessions attended by participants depended on the number of sessions covered by their employer, as well as on personal preferences and their level of need.

Additionally, all participants had unlimited access to a digital library of mental health programs and resources that they could access at any time. These resources included mindfulness and meditation exercises, interactive programs, mental health educational podcasts, self-paced structured educational lessons, and group psychoeducational sessions (called Circles). All digital materials were developed and designed with the support of mental health experts, including an in-house team of clinical psychologists. Digital health programs were designed to cover topics such as emotions, relationships, professional life, healthy lifestyles, and finances. Group sessions included live and recorded sessions led by coaches and therapists that were psychoeducational and addressed a range of health and well-being topics. Data on attendance or utilization of group sessions was not available.

### Measures

#### Demographics

Participants self-reported their age in years. Gender identity was assessed via a multi-select dropdown menu with the following options: Agender, Genderqueer or genderfluid, Māhū, Man, Muxe, Non-binary, Questioning or unsure, Two-spirit, Woman, Prefer to self-describe (with an open-text field), and Prefer not to say. For descriptive purposes, responses other than “Woman” and “Man” were grouped into a “Gender Non-Binary” category. Race and ethnicity were reported using a multi-select dropdown with the following options: American Indian or Alaska Native, Asian or Asian American, Black or African American, Hispanic, Latino, or Spanish origin, White (not Hispanic or Latino), Prefer to self-describe (open-text), and Prefer not to say. Educational attainment was reported via dropdown with the following categories: High school or equivalent, Vocational/Technical school, Bachelor’s degree, Master’s degree, Doctoral degree, and Professional degree. For descriptive analyses, education levels were collapsed into three categories: “< Bachelor’s” (high school or vocational/technical school), “Bachelor’s,” and “>Bachelor’s” (master’s, doctoral, or professional degrees).

#### Depressive Symptoms

We used the 9-item Patient Health Questionnaire-9 (PHQ-9; (Kroenke et al., 2001) to assess depressive symptoms over the past two weeks at baseline and 3-month follow-up. Participants responded on a 4-point scale (0= ‘Not at all’, 3= ‘Nearly every day’) and total scores were summed (range: 0–27), with higher scores indicating higher severity of depression symptomatology. A score ≥ 10 indicates a positive screen for depressive symptoms (Kroenke et al., 2001). The Cronbach alpha was ɑ = 0.85 at baseline and ɑ = 0.83 at 3-month follow-up.

#### Anxiety Symptoms

We used the 7-item Generalized Anxiety Disorder Questionnaire (GAD-7; (Spitzer et al., 2006) to assess anxiety symptoms over the past two weeks at baseline and 3-month follow-up. Participants responded on a 4-point scale (0= ‘Not at all’, 3= ‘Nearly every day’) and total scores were summed (range: 0–21), with higher scores indicating greater severity of anxiety symptomatology. A score ≥ 8 indicates a positive screen for anxiety symptoms (Plummer et al., 2016). The Cronbach’s alpha was ɑ = 0.90 at both baseline and at 3-month follow-up.

#### Distress Tolerance

We used the 15-item Distress Tolerance Scale (DTS; (Simons & Gaher, 2005) to assess participants’ capacity to withstand distress, negative affect, or other aversive psychological states at baseline and 3-month follow-up. Participants responded on a 5-point scale (1= ‘Strongly agree’, 5= ‘Strongly disagree’). After reverse scoring item 6, total scores were summed (range: 15–75), with higher scores indicating higher distress tolerance. The Cronbach’s alpha was *ɑ* = 0.91 at baseline and *ɑ* = 0.92 at 3-month follow-up.

#### Perceived Stress

We used the 4-item Perceived Stress Scale (PSS-4; (Cohen et al., 1983) to assess participants’ level of psychological stress perceived during the past month at baseline and 3-month follow-up. Participants responded on a 5-point scale (0= ‘Never’, 4= ‘Very Often’) and after reverse scoring items 2 and 3, total scores were summed (range: 0–16), with higher scores indicating higher perceived stress. The Cronbach’s alpha was *ɑ* = 0.74 at both baseline and at 3-month follow-up.

#### Self-Compassion

We used the 12-item Self Compassion Scale Short Form (SCS-SF; (Raes et al., 2011) to assess participant’s level of self-compassion at baseline and 3-month follow-up. Participants responded on a 5-point scale (1= ‘Almost never’, 5= ‘Almost always’). The average of each subscale was computed, and the negative subscales ‘self-judgement’, ‘isolation’, and ‘over-identification’ were reverse scored. A total mean score was computed by averaging the subscale means (range: 1–5), with higher scores indicating greater self-compassion. The Cronbach’s alpha was *ɑ* = 0.83 at baseline and *ɑ* = 0.86 at 3-month follow-up.

#### Mindfulness

We used the 15-item Five Facet Mindfulness Questionnaire (FFMQ-15; (Gu et al., 2016) to assess participant’s level of mindfulness at baseline and 3-month follow-up. Participants rated how true each statement was for them on a 5-point scale (1= ‘Never or very rarely true’, 5= ‘Very often or always true’), excluding items from the ‘observe’ subscale. After reverse scoring items 2, 3, 5, 6, 7, 10 and 11, total scores were summed (range: 12–60), with higher scores indicating higher trait mindfulness. The Cronbach’s alpha was *ɑ* = 0.80 at baseline and *ɑ* = 0.83 at 3-month follow-up.

#### Care Modality Preference

During platform registration, participants’ care modality preferences were assessed through the prompt: “When it comes to improving my mental health, I prefer to work…”. Participants could choose one of the following response options: “on my own”, “with a small group”, “one-on-one”, or “I’m not sure”.

#### Primary Topic of Focus

During platform registration, participants were able to choose from over forty topics of focus, or reasons for registering for the platform, during the onboarding process. These topics were wide-ranging and organized by the following well-being dimensions: “my emotions”, “my professional life”, “my physical well-being”, “my relationships”, and “my finances”. After choosing their topics of focus, individuals were prompted to choose one primary topic to focus on first.

#### Care Recommendation

After completing the initial clinical assessment, a proprietary algorithm that factors in participants’ results to the clinical assessments and their care preferences recommended participants a care pathway to start with, including therapy, coaching, or digital programs.

#### Components of Therapeutic Alliance

After each session with a coach, participants had the option to respond to two adapted items from the Working Alliance Inventory (Horvath & Greenberg, 1989) representing the agreement on goals and bond components of therapeutic alliance, respectively: “[My provider] and I are working on agreed upon goals” and “I am confident in [my provider’s] ability to help me”. Participants responded on a five-point scale (1 = Strongly Disagree; 5 = Strongly Agree). These response options were adapted from the Working Alliance Inventory’s original seven-point scale (1 = Never; 7 = Always). The number of survey items and response options was truncated to facilitate survey completion and encourage higher response rates from participants in a real-world setting. One score was computed for each participant with available data by averaging each participant’s response to these two items across all sessions within the 3-month follow-up period. This assessment has shown appropriate reliability and validity previously (Sagui-Henson et al., 2022), with a Cronbach alpha of 0.72.

#### Platform Engagement

To assess engagement in the platform, we calculated the total number of therapy and coaching sessions completed and the number of self-guided digital resources used during the 3-month study period. We did not have available data on group psychoeducation session engagement as participation was anonymous.

### Statistical Analyses

We conducted analyses using R version 4.0.4 (R Core Team, 2024). We conducted analyses in the overall sample of coaching users and stratified by baseline clinical status (“elevated risk” = baseline PHQ-9 ≥ 10 and/or GAD-7 ≥ 8, and “lower risk” = baseline PHQ-9 < 10 and/or GAD-7 < 8). Descriptive statistics were conducted to evaluate topics of focus, care recommendations, and engagement.

Our aims were to evaluate longitudinal changes in (1) transdiagnostic emotional processes, including distress tolerance, perceived stress, self-compassion, and mindfulness and (2) mental health outcomes, including depressive and anxiety symptoms. We first performed paired samples t-tests to measure changes in each outcome from participants’ baseline to 3-month follow-up assessment. We conducted these in the overall sample of coaching users and in each clinical risk group (elevated risk and lower risk). We also constructed linear regressions to test whether changes in outcomes differed by baseline clinical status. In the regressions, the predictor was the binary baseline clinical status variable (0 = lower risk, 1 = elevated risk) and the outcome was the difference between the baseline and 3-month follow-up score for each emotional process or mental health outcome.

Finally, we examined the percentage of participants who reported various changes in depressive and anxiety symptom scores and severity. We looked at these changes separately by baseline clinical risk status because, from a population health perspective, each group has different treatment goals (elevated risk users have the goal of improving and lower risk users have the goal of maintaining good mental health). Maintenance in the lower-risk group reflects a meaningful outcome: preserving good mental health and building skills that reduce future risk. This aligns with broader public health models of prevention and early intervention.

For both baseline risk groups, we examined whether participants’ clinical score improved (score decreased by a clinically meaningful amount, defined as 5 + points for PHQ-9 (Kroenke, 2012) or 4 + points for GAD-7 (Plummer et al., 2016), clinical score maintained (score did not increase or decrease by a clinically meaningful amount), and clinical score worsened (score increased by a clinically meaningful amount).

For elevated risk users, we examined whether participants’ clinical severity recovered (score started above the clinical cut-off [≥ 10 for PHQ-9, ≥ 8 for GAD-7] and ended below the cut-off) or clinical severity maintained (score started and ended above the clinical cut-off). We also looked at a combined metric of whether their clinical score improved and/or clinical severity recovered (met criteria for one or both metrics). For lower risk users, we looked at whether participants’ clinical severity maintained (score started and ended below the clinical cut-off) or clinical severity worsened (score started below the clinical cut-off and ended above the cut-off). To be comprehensive, we reported the changes separately by depressive and anxiety symptoms and combined.

## Results

### Study Participants

Out of 8,786 individuals outreached for the study, 2,032 (23.1%) completed the screening form, 1,193 (13.6%) provided consent, and 950 (10.8%) enrolled and completed the baseline survey. Participants were employed across a range of industries, including technology (61.3%), care-related sectors such as healthcare and education (11.9%), labor-intensive fields such as manufacturing and transportation (8.5%), service industries such as hospitality and food service (7.4%), and other sectors not otherwise classified (10.9%). Of the 950 who enrolled, 703 (74%) completed the 3-month follow-up survey. Of the 703 with follow-up data, 532 participants engaged with at least one service. There were no significant differences in demographic characteristics or on any outcome measured at baseline between people who did not engage with anything on the Modern Health app after baseline (*n* = 171) and those that engaged (*n* = 532) (*p*s > 0.05, see Supplementary Tables 1–2).

Among the 532 participants who engaged with care between baseline and follow-up, 366 (68.8%) were recommended coaching and 271 (74%) of those recommended went on to use coaching services. In total during the study period, 302 (56.8%) participants engaged in at least one coaching session, 165 (31%) used at least one therapy session, and 362 (68%) used at least one digital resource.

To focus the analysis on participants for whom coaching was the primary modality of care, we identified a subsample of 266 coaching-majority users. These participants either used coaching alone or used coaching in combination with other services in a way that reflected coaching as their principal form of care. Specifically, participants who used coaching alongside digital resources were retained in this sample, as digital tools are intended to supplement, rather than replace, provider-delivered care. Participants who used both coaching and therapy were retained only if they attended more coaching sessions than therapy sessions. Those who engaged in more therapy than coaching (*n* = 36) were excluded to ensure that the analytic sample reflected a coaching-majority use pattern.

All primary outcome analyses were conducted in this revised sample (*n* = 266). Participants in the analytic sample (*n* = 266) were 33 years old (*SD* = 7.92), 62.4% identified as a woman, and 40.2% identified as a person of color (see Table 1 for sample demographics and characteristics).

**Table 1:** Demographic characteristics, topics of focus, and care recommendations in the overall sample and by baseline clinical risk

	Overall Coaching Users (*n*=266)	Elevated Risk Coaching Users (*n*=113)	Lower Risk Coaching Users (*n*=153)
Demographic Characteristics			
Age,*M* (*SD*)	33.49 (7.92)	32.92 (7.89)	33.92 (7.94)
Gender Identity,*n* (%)			
Woman	166 (62.4)	78 (69.0)	88 (57.5)
Man	91 (34.2)	29 (25.7)	62 (40.5)
Gender non-binary	9 (3.9)	6 (5.3)	3 (2.0)
Race/Ethnicity,*n* (%)			
Asian or Asian American	41 (15.4)	14 (12.4)	27 (17.6)
Black or African American	18 (6.8)	10 (8.87.4)	8 (5.2)
Hispanic, Latino, or Spanish Origin	25 (9.4)	9 (8.07.4)	16 (10.5)
Non-Hispanic White	159 (59.8)	70 (61.9)	89 (58.2)
More than 1 race/ethnicity	23 (8.6)	10 (8.8)	13 (8.5)
Education,*n* (%)			
<Bachelors	77 (28.9)	26 (23.0)	51 (33.3)
Bachelors	160 (60.2)	71 (62.8)	89 (58.2)
>Bachelors	29 (10.9)	16 (14.2)	13 (8.5)
Topics of Focus,*n* (%)			
My emotions	130 (48.9)	63 (55.8)	67 (43.8)
My finances	10 (3.8)	3 (2.7)	7 (4.6)
My physical well-being	27 (10.2)	15 (13.3)	12 (7.8)
My professional life	44 (16.5)	15 (13.3)	29 (19.0)
My relationships	55 (20.7)	17 (15.0)	38 (24.8)
Care Recommendation,*n* (%)*			
Coaching	245 (92.1)	104 (92.0)	141 (92.2)
Therapy	6 (2.3)	5 (4.4)	1 (0.7)
Digital Resources	13 (4.9)	4 (3.5)	9 (5.9)

Note. M = mean, SD = standard deviation. Elevated risk for depressive or anxiety symptoms was defined as baseline depressive or anxiety symptom scores above the clinical cut-off (≥ 10 for depressive symptoms, ≥ 8 for anxiety symptoms). Lower risk for depressive or anxiety symptoms was defined as baseline depressive or anxiety symptom scores below the clinical cut-off ( < 10 for depressive symptoms, < 8 for anxiety symptoms).*Difference is statistically significant (*p <* .05)

### Descriptive Data

**Fig. 1:**
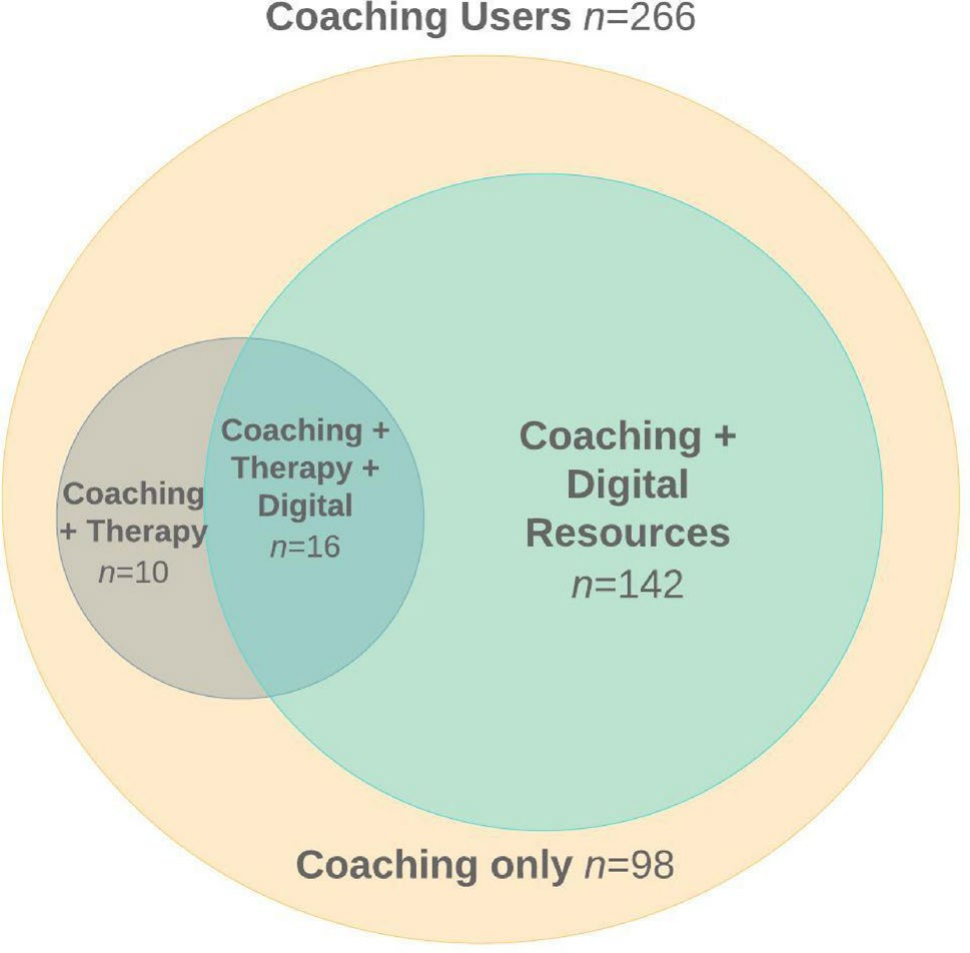
Platform engagement among coaching users (*n* = 266). All users engaged with coaching as their primary modality of care

See Tables 1 and 2 for descriptive results. Regarding care recommendations, among coaching users (*n* = 266), 245 (92.1%) were recommended coaching and 21 (7.9%) were recommended another service (13 digital, 6 therapy, 2 group sessions) but used coaching as their primary care modality. Regarding service use, 98 (36.8%) used coaching only and 168 (63.2%) used coaching as their primary modality of care plus at least one other service (see Fig. 1). In the coaching-plus group, 10 (6.0%) participants used coaching plus therapy, 142 (84.5%) used coaching plus digital resources, and 16 (9.5%) used coaching plus therapy and digital resources. There were no significant differences between the coaching-only and coaching-plus groups on any demographic or baseline clinical factor, including age, gender identity, race/ethnicity, education, or baseline scores for depressive or anxiety symptoms, distress tolerance, perceived stress, self-compassion, or mindfulness (*p*s > 0.05).

Regarding the number of coaches delivering care, 254 participants worked with a single coach and 12 participants worked with two different coaches during the study period. Thus, 278 total coaching relationships occurred. Almost half (48.9%) of all coaching users selected “my emotions” as their primary topic of focus. Therapeutic alliance data were available for 145 participants: the mean alliance rating was 4.54 (*SD* = 0.58), median = 4.75, range = 2–5, and 129 (89%) reported a therapeutic alliance rating ≥ 4.

Overall, participants engaged in an average of 2.52 (*SD* = 1.97) coaching sessions over three months (see Table 2). Coaching-only users (*n* = 98) engaged in an average of 2.06 (*SD* = 1.62) coaching sessions and coaching-plus users (*n* = 168) engaged in an average of 2.78 (*SD* = 2.11 (coaching sessions). Full platform engagement data in the coaching-only and coaching-plus groups can be found in Supplementary Table 3. Average depressive and anxiety symptom scores in the overall sample were below the clinical cut-offs for the PHQ-9 (score of 10) and GAD-7 (score of 8). As expected, given the stratified care model, elevated risk participants (*n* = 113) had average baseline depressive (*M* = 11.41, *SD* = 4.29) and anxiety symptom scores (*M* = 10.77, *SD* = 4.25) below the cut-off of 15 for severe depression or anxiety.

**Table 2:** Platform engagement and baseline clinical factors in the overall sample and by baseline clinical risk

	Overall Coaching Users (*n*=266)	Elevated Risk Coaching Users (*n*=113)	Lower Risk Coaching Users (*n*=153)
Platform Engagement,*M* (*SD*)			
Coaching Sessions*	2.52 (1.97)	2.84 (2.29)	2.28 (1.66)
Therapy Sessions*	0.20 (0.77)	0.37 (1.10)	0.07 (0.33)
Digital Resources	2.07 (3.17)	2.27 (3.36)	1.93 (3.04)
Clinical Factors,*M* (*SD*)			
Baseline Depressive Symptoms*	7.33 (4.96)	11.41 (4.29)	4.32 (2.80)
Baseline Anxiety Symptoms*	6.68 (4.79)	10.77 (4.25)	3.65 (2.25)

Note. M = mean, SD = standard deviation. Possible ranges for scores include 0–27 for depressive symptoms and 0–21 for anxiety symptoms. Elevated risk for depressive or anxiety symptoms was defined as baseline depressive or anxiety symptom scores above the clinical cut-off (≥ 10 for depressive symptoms, ≥ 8 for anxiety symptoms). Lower risk for depressive or anxiety symptoms was defined as baseline depressive or anxiety symptom scores below the clinical cut-off (< 10 for depressive symptoms, < 8 for anxiety symptoms).

*Difference is statistically significant (*p*<.05)

### Changes in Transdiagnostic Emotional Processes

Overall, coaching users reported statistically significant improvements in transdiagnostic emotional processes, including distress tolerance (+ 4.1%), perceived stress (−8.4%), self-compassion (+ 5.9%), and mindfulness (+ 2.6%) (see Table 3). Distress tolerance statistically significantly increased in both elevated risk (+ 4.7%) and lower risk users (+ 3.7%), yet the difference in change between these two groups was not significant (*b* = 0.18, *F*(1, 262) = 0.29, *p* =.86, *R*^*2*^ = 0.001). Elevated risk users reported a statistically significant reduction in perceived stress (−10.0%), while lower risk users reported no significant change, yet this difference in change between groups was not significant (*b* = 0.46, *F*(1, 262) = 2.31, *p* =.13, *R*^*2*^ = 0.01). Self-compassion statistically significantly increased in both elevated risk (+ 7.6%) and lower risk users (+ 5.2%), and the difference in change between these two groups was not significant (*b* = 0.04, *F*(1, 260) = 0.63, *p* =.43, *R*^*2*^ = 0.002). Elevated risk users reported a statistically significant increase in mindfulness (+ 3.3%) as did lower risk users (+ 2.1%), yet this difference in change between groups was not significant (*b* = 0.33, *F*(1, 260) = 0.24, *p* =.62, *R*^*2*^ = 0.001). To assess whether outcomes differed by type of care received, we conducted post-hoc regression analyses comparing coaching-only (*n* = 98) and coaching-plus (*n* = 168) users and found no significant differences in changes in any transdiagnostic emotional process (*p*s < 0.05).

**Table 3:** Paired-samples t test results for transdiagnostic emotional processes in the overall sample and by baseline clinical risk

Sample	Baseline	Follow-up	Diff	95% CIs	*t* (*df*)	*p*	Cohen*d*	% change
	M (SD)	M (SD)	M					
Overall (*n*=266)								
Distress Tolerance	49.66 (11.73)	51.71 (12.25)	2.02	1.01, 3.04	3.93 (263)	<0.001	0.17	+ 4.1%
Perceived Stress	6.56 (2.49)	6.04 (2.64)	−0.55	−0.85, −0.25	3.63 (263)	<0.001	0.21	−8.4%
Self-Compassion	2.90 (0.58)	3.08 (0.65)	0.17	0.12, 0.23	6.27 (261)	<0.001	0.28	+ 5.9%
Mindfulness	39.36 (6.34)	40.34 (6.93)	1.02	0.36, 1.68	3.06 (261)	0.002	0.15	+ 2.6%
Elevated Risk (*n*=113)								
Distress Tolerance	45.30 (9.76)	47.42 (11.15)	2.12	0.62, 3.63	2.80 (112)	0.006	0.20	+ 4.7%
Perceived Stress	8.07 (2.15)	7.26 (2.40)	−0.81	−1.22, −0.41	3.98 (112)	<0.001	0.36	−10.0%
Self-Compassion	2.64 (0.48)	2.84 (0.58)	0.20	0.12, 0.28	5.03 (111)	<0.001	0.37	+ 7.6%
Mindfulness	36.54 (6.19)	37.72 (6.47)	1.21	0.16, 2.27	2.28 (111)	0.02	0.19	+ 3.3%
Lower Risk (*n*=153)								
Distress Tolerance	52.88 (12.04)	54.91 (12.09)	1.95	0.56, 3.33	2.78 (150)	0.006	0.16	+ 3.7%
Perceived Stress	5.45 (2.13)	5.13 (2.44)	−0.35	−0.78, 0.07	1.63 (150)	0.10	0.15	−6.4%
Self-Compassion	3.10 (0.57)	3.25 (0.64)	0.16	0.08, 0.23	4.04 (149)	<0.001	0.25	+ 5.2%
Mindfulness	41.44 (5.61)	42.30 (6.63)	0.88	0.03, 1.73	2.04 (149)	0.04	0.14	+ 2.1%

Note. M = mean, SD = standard deviation, CI = confidence interval. Possible ranges for scores include 15–75 for distress tolerance, 0–16 for perceived stress, 1–5 for self-compassion, and 12–60 for mindfulness. Elevated risk for depressive or anxiety symptoms was defined as baseline depressive or anxiety symptom scores above the clinical cut-off (≥ 10 for depressive symptoms, ≥ 8 for anxiety symptoms). Lower risk for depressive or anxiety symptoms was defined as baseline depressive or anxiety symptom scores below the clinical cut-off ( < 10 for depressive symptoms, < 8 for anxiety symptoms)

### Changes in Depressive and Anxiety Symptoms

Overall, coaching users reported significant reductions in depressive (−22.5%) and anxiety (−12.0%) symptoms with small to medium effect sizes (see Table 4). Elevated risk users reported a statistically significant reduction in depressive symptoms (−30.3%), while lower risk users reported no significant change, and this difference in change between groups was statistically significant (*b* = −3.15, *F*(1, 263) = 37.91, *p* <.001, *R*^*2*^ = 0.13). Thus, elevated risk users reported significantly greater reductions in depressive symptoms compared to lower risk users. Elevated risk users reported a statistically significant reduction in anxiety (−21.3%), while lower risk users reported no significant change, and this difference in change between groups was statistically significant (*b* = −2.61, *F*(1, 262) = 23.98, *p* <.001, *R*^*2*^ = 0.08). Thus, elevated risk users reported significantly greater reductions in anxiety compared to lower risk users. To assess whether outcomes differed by type of care received, we ran post-hoc regression analyses comparing coaching-only (*n* = 98) and coaching-plus (*n* = 168) users and found no significant differences in changes in either depressive or anxiety symptom outcomes (*p*s < 0.05).

**Table 4:** Paired-samples t test results for transdiagnostic emotional processes in the overall sample and by baseline clinical risk

Sample	Baseline	Follow-up	Diff	95% CIs	*t* (*df*)	*p*	Cohen*d*	% change
	M (SD)	M (SD)	M					
Overall (*n*=266)								
Depressive Symptoms	7.33 (4.96)	5.71 (4.15)	−1.65	−2.18, −1.12	6.12 (264)	0.001	0.36	−22.5%
Anxiety Symptoms	6.68 (4.79)	5.92 (4.75)	− 0.80	−1.34, −0.26	2.91 (263)	0.01	0.17	−12.0%
Elevated Risk (*n*=113)								
Depressive Symptoms	11.41 (4.29)	7.95 (4.13)	−3.46	−4.32, −2.60	7.93 (112)	0.001	0.82	−30.3%
Anxiety Symptoms	10.77 (4.25)	8.48 (4.97)	−2.29	−3.25, −1.34	4.76 (112)	0.001	0.49	−21.3%
Lower Risk (*n*=153)								
Depressive Symptoms	4.32 (2.80)	4.04 (3.30)	−0.31	−0.90, 0.28	1.03 (151)	0.30	0.10	−7.2%
Anxiety Symptoms	3.65 (2.25)	4.01 (3.54)	0.32	−0.25, 0.89	1.10 (151)	0.27	0.11	+ 8.8%

Note. M = mean, SD = standard deviation, CI = confidence interval. Possible ranges for scores include 15–75 for distress tolerance, 0–16 for perceived stress, 1–5 for self-compassion, and 12–60 for mindfulness. Elevated risk for depressive or anxiety symptoms was defined as baseline depressive or anxiety symptom scores above the clinical cut-off (≥ 10 for depressive symptoms, ≥ 8 for anxiety symptoms). Lower risk for depressive or anxiety symptoms was defined as baseline depressive or anxiety symptom scores below the clinical cut-off ( 10 for depressive symptoms, 8 for anxiety symptoms).

Regarding changes in depressive and anxiety symptom scores and severity among elevated risk coaching users (see Table 5), 69 (61.1%) reported their clinical score improved, 74 (65.5%) reported their clinical severity recovered, and 81 (71.7%) reported their clinical score improved and/or their clinical severity recovered. Regarding changes among lower risk coaching users (see Table 6), 101 (66%) reported their clinical score maintained and 147 (96.1%) reported their clinical severity maintained.

**Table 5:** Changes in depressive and anxiety symptom scores and severity among elevated risk coaching users (n = 113)

Outcome Category	Elevated Depressive Symptoms (***n***=79) *n* (%)	Elevated Anxiety Symptoms (***n***=93) *n* (%)	Elevated Depressive and/or Anxiety Symptoms (***n***=113) *n* (%)
Clinical Score Change			
Clinical score improved	39 (49.4)	46 (49.5)	69 (61.1)
Clinical score maintained	39 (49.4)	38 (40.9)	25 (22.1)
Clinical score worsened	1 (1.3)	9 (9.7)	19 (16.8)
Clinical Severity Change			
Clinical severity recovered	47 (59.5)	46 (49.5)	74 (65.5)
Clinical severity maintained	32 (40.5)	47 (50.5)	39 (34.5)
Clinical Score and Severity Change			
Clinical score improved and/or clinical severity recovered	52 (65.8)	55 (59.1)	81 (71.7)

Note. Depressive symptoms were assessed with the Patient Health Questionnaire-9 (PHQ-9) and anxiety symptoms were assessed with the Generalized Anxiety Disorder Questionnaire-7 (GAD-7)

Elevated risk for depressive or anxiety symptoms was defined as baseline depressive or anxiety symptom scores above the clinical cut-off (≥ 10 for PHQ-9, ≥ 8 for GAD-7)

The following definitions were used to determine clinical change: Clinical score improved = score decreased by a clinically meaningful amount (5 + points for PHQ-9 and 4 + points for GAD-7); Clinical score maintained = score did not increase or decrease by a clinically meaningful amount; Clinical score worsened = score increased by a clinically meaningful amount; Clinical severity recovered = score started above the clinical cut-off and ended below the cut-off; Clinical severity maintained = score started and ended above the clinical cut-off; Clinical score improved and/or clinical severity recovered = participant met criteria for one or both metrics

**Table 6:** Changes in depressive and anxiety symptom scores and severity among lower risk coaching users (n = 153)

Outcome Category	Lower Depressive Symptoms (***n***=186) *n* (%)	Lower Anxiety Symptoms (***n***=173) *n* (%)	Lower Depressive and Anxiety Symptoms (***n***=153) *n* (%)
Clinical Score Change			
Clinical score improved	20 (10.8)	22 (12.7)	27 (17.6)
Clinical score maintained	150 (80.6)	124 (71.7)	101 (66.0)
Clinical score worsened	16 (8.6)	25 (14.5)	23 (15.0)
Clinical Severity Change			
Clinical severity maintained	169 (90.9)	146 (84.4)	147 (96.1)
Clinical severity worsened	17 (9.1)	25 (14.5)	5 (3.3)

Note. Depressive symptoms were assessed with the Patient Health Questionnaire-9 (PHQ-9) and anxiety symptoms were assessed with the Generalized Anxiety Disorder Questionnaire-7 (GAD-7)

Lower risk for depressive or anxiety symptoms was defined as baseline depressive or anxiety symptom scores below the clinical cut-off (≥ 10 for PHQ-9, ≥ 8 for GAD-7)

The following definitions were used to determine clinical change: Clinical score improved = score decreased by a clinically meaningful amount (5 + points for PHQ-9 and 4 + points for GAD-7); Clinical score maintained = score did not increase or decrease by a clinically meaningful amount; Clinical score worsened = score increased by a clinically meaningful amount; Clinical severity maintained = score started and ended below the clinical cut-off; Clinical severity worsened = score started below the clinical cut-off and ended above the cut-off

## Discussion

This study evaluated the impact of evidence-based, technology-enabled coaching services, delivered as part of a blended care digital mental health platform, on transdiagnostic emotional processes and clinical outcomes in a diverse sample of working adults in the US. Overall, the findings demonstrate that people who engaged with coaching as their primary care modality reported significant improvements in emotion regulation skills and mental health symptoms. Participants starting care with higher baseline depressive or anxiety symptoms (but still, on average, below the cut-off for severe symptoms) reported greater improvements, suggesting that scaling up services for mental health through coaching can be especially effective in people with elevated needs.

Participants showed statistically significant gains in transdiagnostic processes such as distress tolerance, mindfulness, and self-compassion. These are critical emotion regulation skills that underlie many common mental health conditions, and targeting them may help individuals better manage stress, reduce self-criticism, and build emotional resilience (Krueger & Eaton, 2015). The observed reductions in depressive and anxiety symptoms, by approximately 22% and 12%, respectively, further underscore the clinical relevance of these improvements. These findings support the potential of coaching within a blended care model to serve as an intervention that addresses foundational psychological mechanisms and promotes overall well-being.

To account for the diverse needs of users on this employer-sponsored platform, we adopted a population health framework and stratified our analyses by baseline clinical risk. Because the platform is accessible to individuals across the full continuum of symptom severity, including many who present with mild or subclinical symptoms, this stratification allowed us to examine how coaching impacted users with distinct care goals. While elevated risk individuals may seek symptom reduction, lower-risk users often engage with coaching to maintain mental health and build emotional resilience. Distinguishing between these groups provides a clearer understanding of how coaching supports both preventive and responsive care.

Although t-tests revealed that elevated risk users showed significant improvements in transdiagnostic emotional skills while lower-risk users generally showed no significant change, regression analyses indicated no significant differences in change scores between the two groups. This suggests that coaching may support similar improvements in emotional processes across varying levels of baseline clinical risk. These findings reinforce the idea that coaching is a flexible and effective intervention for a broad spectrum of mental health needs. Notably, many elevated risk users in this study had moderate symptom levels that were below the threshold for severe distress. These individuals may not yet meet diagnostic criteria or access traditional clinical care. By targeting transdiagnostic mechanisms such as distress tolerance and self-compassion across all baseline risk levels, coaching may serve as a critical intervention to improve well-being and emotional functioning in individuals without a formal diagnosis.

Our results did show that elevated risk users experienced significantly greater reductions in depressive (30%) and anxiety (21%) symptoms compared to lower risk users who maintained their low symptom levels. Nearly 72% of elevated risk participants improved or recovered from clinical levels of depressive or anxiety symptoms during the study period, with comparatively low rates of clinical score maintenance (34.5%) and worsening (17%). It is estimated that 38% of US adults with depressive symptoms had moderate to severe levels in 2019 (Centers for Disease Control and Prevention, 2020), representing 7% of the population. This is a large group either not receiving treatment or currently relying on specialized, highly trained individuals to receive care. Although coaching may have previously been considered an intervention for people with lower clinical acuity (e.g., career or wellness coaching), our results suggest that coaches, who can be trained more efficiently, can deliver interventions feasibly and effectively to people with higher mental health needs.

Among lower risk coaching users, 96% maintained low depressive and anxiety symptoms, and only 15% reported their score worsened a clinically meaningful amount during the study period. These results reinforce the preventive potential of coaching, particularly in the context of a stratified care model designed to deliver appropriate interventions based on individual need (Delgadillo et al., 2022). Preventive interventions that promote emotional regulation, self-awareness, and coping skills are essential for reducing the long-term burden of mental health conditions. By supporting maintenance of well-being, coaching may help mitigate the escalation of symptoms and potentially reduce the need for more intensive services over time. From both a public health and service delivery perspective, this dual role (i.e. supporting symptom reduction in higher risk individuals and maintenance in lower risk ones) makes coaching a valuable asset within stratified, blended care systems.

Moreover, by triaging lower and moderate risk individuals to coaching, higher intensity resources such as therapy and psychiatry can be preserved for those with the most complex needs. This is particularly important given growing concerns around mental health workforce shortages and the rising cost of care in the US. Coaching services, which are often two to three times less expensive than traditional therapy (Prescott et al., 2022), may also offer a cost-effective solution for supporting individuals with subclinical or moderate symptoms. Ultimately, scalable coaching interventions have the potential to not only improve outcomes across risk groups but also contribute to more sustainable healthcare delivery.

It is also important to note that this was a real-world evidence study. Rather than altering the platform or controlling care delivery, we assessed clinical outcomes as they occurred naturally through routine engagement with services. All participants used coaching as their primary modality, but some also engaged with other components of the platform, such as therapy or digital tools. We conducted sensitivity analyses to evaluate whether outcomes differed between coaching-only users and those who used coaching alongside other services and found no significant differences in emotional process or mental health outcomes. These findings support the flexibility and adaptability of coaching within a blended care model. Importantly, they suggest that coaching can be effective whether delivered alone, or as the primary form of care in combination with other services.

These findings have important implications for expanding the reach and efficiency of mental health care. Coaching can help fill critical gaps by supporting individuals with elevated symptoms who may not require or access traditional therapy, as well as by providing timely, preventive care for lower-risk individuals. By equipping paraprofessionals with evidence-based skills to address transdiagnostic mechanisms, coaching can extend the mental health workforce and reserve specialized clinical resources for those with the most severe needs. This study adds to a growing body of literature supporting the effectiveness of coaching in improving both clinical symptoms and foundational emotional processes (Roos et al., 2024; Sagui Henson et al., 2024; Wu et al., 2021), reinforcing its value as a scalable, flexible intervention within a population health framework.

Second, integrating coaching into a comprehensive digital platform allows for the scalable, efficient delivery of high-quality care. Digital platforms reduce traditional barriers to care, such as geographic limitations, stigma, and scheduling constraints, thereby supporting broader implementation of evidence-based interventions (Kazdin & Blase, 2011). Further, one of the reasons coaching can be so broadly applicable is because it is not limited by state-based licensing requirements. These platforms also allow for technology-enabled safety and quality features. For example, therapeutic alliance data showed a mean rating of 4.54 (out of 5), with 89% rating their coach alliance ≥ 4, indicating strong positive provider relationships. Our results suggest that leveraging digital tools for healthcare delivery (e.g., intake assessments, care recommendations, provider matching, provider network management, telehealth visits, and quality monitoring) to deploy coaching services can play a pivotal role in mitigating the mental health treatment gap, particularly for underserved populations. Using technology-enabled services increases access to the right care at the right time and is a vehicle through which coaching can have the biggest impact on outcomes at scale.

### Strengths and Limitations

There are several notable strengths of the current investigation, including the use of real-world data. This allowed us to observe how users engaged with the blended services in the digital mental health platform in an ecologically valid way and how they used coaching services in their daily lives. This approach highlights the efficient delivery and scalability of digital coaching services in real-life settings. Another key strength includes the diversity of the sample, which encompasses a wide range of ages, ethnicities, and education levels. This diversity suggests that coaching services can help users across a range of clinical needs navigate a variety of daily challenges. Further, coaching may be a more accessible and less stigmatized entry point into effective mental health support for people across a range of demographic groups. The prospective study design also allowed us to examine the impact of coaching services over time through robust pre-post analyses. This approach allowed us to capture baseline levels prior to the initiation of coaching services and make direct comparisons after three months. Tracking these pre-post changes provided us with strong evidence that coaching services may reduce symptoms of depressive and anxiety symptoms and may be especially useful for individuals starting out with elevated symptoms.

The findings in this study should also be interpreted in light of several limitations. First, as all participants were eligible to receive the digital mental health services, the study lacked a control group for comparison. Therefore, we are unable to make causal inferences about the effect of platform engagement. Future research should explore the comparative effectiveness of coaching relative to treatment as usual or to other modalities and examine the mechanisms underlying observed improvements. Next, participants self-reported their mental health symptomatology and transdiagnostic emotional processes. Recall bias may have limited participants’ ability to accurately evaluate themselves and recall their experiences from over the past few weeks. Additionally, participants may have responded in a way they believed to be socially desirable.

Further, we did not have access to data on the content of coaching sessions or fidelity to coaching protocols. The platform delivers real-world care by connecting people with qualified coaches as part of their routine employer-sponsored benefits. Although coaches in the platform were trained in and vetted for using evidence-based practices, received ongoing training and consultation, and therapeutic alliance ratings by participants were high, suggesting excellent patient satisfaction with care, future research should venture to obtain fidelity data to better understand the relative contribution of specific evidence-based strategies to observed outcomes. Next, we were unable to include detailed demographic or background information about the coaches, limiting our ability to assess how provider-level characteristics may have influenced participant outcomes.

Another important limitation is the lack of longer-term follow-up beyond three months. While participants reported meaningful short-term improvements, the durability of these gains is unknown. Future studies should examine whether improvements in emotional processes and symptoms are sustained over time and whether ongoing coaching engagement is necessary to maintain these benefits. Finally, while this study focused on users who identified coaching as their primary care modality, some participants also engaged with other services on the platform. Although our analyses suggest no significant differences in outcomes between coaching-only and coaching-plus groups, it remains possible that concurrent care contributed to outcomes in ways we could not fully disentangle.

## Conclusions

This study demonstrates that certified coaches who are trained in evidence-based practices, but not licensed in mental health care, can deliver effective interventions that improve several important patient-reported outcomes. As research on psychological constructs and treatments continues to evolve, it is clear that addressing mental health concerns at a population level will necessitate the implementation of a variety of strategies. Evidence-based therapies delivered by licensed therapists are not sufficient to address the public health need, nor are they the only interventions with demonstrated efficacy and effectiveness. By redefining who can deliver care and to whom and leveraging technology alongside existing psychological treatment research to scale these efforts, technology-enabled coaching represents a promising, cost-effective solution to bridge the mental health treatment gap and meaningfully impact population health. Future research should continue to explore innovative care models that expand access to high-quality, cost-effective mental health support.

## Supplementary Information

Below is the link to the electronic supplementary material.

Supplementary Material 1

## Data Availability

Individual de-identified data that underlie the results reported in this manuscript can be shared privately for research purposes upon receipt of a methodologically sound proposal, and whose proposed use of the data from the study related to this article are approved by the authors and Modern Health legal and security teams. To gain access, requesters will need to submit a proposal to the corresponding author and sign a data access agreement that includes a commitment to: (1) using the data only for research purposes; (2) not attempt to, or actually, re-identify any individual; (3) securing the data using appropriate safeguards; and (4) destroying or returning the data after analyses are completed.
