# Supplementary material for: Addressing the Gap: Real-World Evidence of Technology-Enabled Coaching Services for Mental Health
Source: Adm Policy Ment Health. 2025 Sep 27;52(6):1311–26. doi: 10.1007/s10488-025-01473-8 (PMC12628405; doi:10.1007/s10488-025-01473-8)
Supplement: Supplementary file 1 — Supplementary Material 1 [file 10488_2025_1473_MOESM1_ESM.docx]

**Supplementary Materials**

For manuscript “Addressing the Gap: Real-World Evidence of Technology-Enabled Coaching Services for Mental Health” (APMH-D-25-00045)

| **Supplementary Table 1**  Demographic Characteristics of Engagers and Non-Engagers | | | | |
| --- | --- | --- | --- | --- |
|  | **Engagers**  (*n* = 532) | **Non-Engagers**  (*n* = 171) | $\boldsymbol{x}$***^2^* or t** | ***p*** |
| **Demographic Characteristics** | |  |  |  |
| **Age**, *M* (*SD*) | 33.87 (8.7) | 33.91 (9.1) | 0.04 | .97 |
| **Gender Identity,** *n* (%) |  |  | 0.54 | .76 |
| Woman | 329 (61.8) | 101 (59.1) |  |  |
| Man | 180 (33.8) | 61 (35.7) |  |  |
| Gender non-binary | 23 (4.3) | 9 (5.3) |  |  |
| **Race/Ethnicity,** *n* (%) |  |  | 10.37 | .11 |
| Asian or Asian American | 93 (17.5) | 41 (24.0) |  |  |
| Black or African American | 35 (6.6) | 3 (1.8) |  |  |
| Hispanic, Latino, or Spanish Origin | 47 (8.8) | 19 (11.1) |  |  |
| Non-Hispanic White | 316 (59.4) | 97 (56.7) |  |  |
| More than 1 race/ethnicity | 38 (7.1) | 11 (6.4) |  |  |
| **Education,** *n* (%) |  |  | 5.90 | .05 |
| <Bachelors | 151 (28.4) | 37 (21.6) |  |  |
| Bachelors | 315 (59.2) | 102 (59.6) |  |  |
| >Bachelors | 66 (12.4) | 32 (18.7) |  |  |
| *Note. n* = 703*. M* = mean, *SD* = standard deviation. There were no significant demographic differences between those who engaged in services and those who did not engage. | | | | |

| **Supplementary Table 2**  Baseline Clinical Score Differences between Engagers and Non-Engagers | | | | |
| --- | --- | --- | --- | --- |
|  | **Engagers**  (*n* = 532) | **Non-Engagers**  (*n* = 171) | $\boldsymbol{x}$***^2^* or t** | ***p*** |
| **Depressive Symptom Screen,** *n* (%) |  |  | 1.20 | .27 |
| Negative Screen | 341 (64.1) | 101 (59.1) |  |  |
| Positive Screen | 191 (35.9) | 70 (40.1) |  |  |
| **Anxiety Symptom Screen,** *n* (%) |  |  | 0.03 | .86 |
| Negative Screen | 308 (57.9) | 101 (59.1) |  |  |
| Positive Screen | 224 (42.1) | 70 (40.1) |  |  |
| **Depressive Symptoms,** *M* (*SD*) | 8.18 (5.50) | 8.44 (5.89) | 0.52 | .61 |
| **Anxiety Symptoms,** *M* (*SD*) | 7.51 (5.33) | 7.36 (5.44) | -0.31 | .75 |
| **Distress Tolerance,** *M* (*SD*) | 48.34 (12.05) | 48.59 (12.91) | 0.22 | .83 |
| **Perceived Stress,** *M* (*SD*) | 6.83 (2.75) | 6.91 (3.01) | 0.33 | .74 |
| **Self-Compassion,** *M* (*SD*) | 2.89 (0.67) | 2.91 (0.74) | 0.49 | .62 |
| **Mindfulness**, *M (SD)* | 38.98 (6.99) | 37.89 (7.14) | -1.74 | .08 |
| *Note.* A positive screen for depressive or anxiety symptoms was defined as baseline depressive or anxiety symptom scores above the clinical cut-off (≥ 10 for depressive symptoms, ≥ 8 for anxiety symptoms). All other variables were measured on a continuous scale. There were no significant differences in baseline variables between those who engaged in services and those who did not engage. | | | | |

| **Supplementary Table 3** | | | |
| --- | --- | --- | --- |
| *Platform Engagement the Overall Sample, Coaching-Only Sample, and Coaching-Plus Sample* | | | |
|  | **Coaching Sessions**  *M* (*SD*) | **Therapy Sessions**  *M* (*SD*) | **Digital Resources**  *M* (*SD*) |
| **Coaching Subsamples**, *M* (*SD*) |  |  |  |
| Overall Coaching Users (*n*=266) | 2.52 (1.97) | 0.20 (0.77) | 2.07 (3.17) |
| Coaching-Only Users (*n*=98) | 2.06 (1.62) | 0 (0) | 0 (0) |
| Coaching-Plus Users (*n*=168) | 2.78 (2.11) | 0.32 (0.96) | 3.28 (3.46) |
| Coaching Plus Therapy (*n*=10) | 4 (3.56) | 2.2 (2.25) | 0 (0) |
| Coaching Plus Digital (*n*=142) | 2.58 (1.80) | 0 (0) | 3.48 (3.36) |
| Coaching Plus Therapy and Digital (*n*=16) | 3.75 (2.98) | 1.94 (1.00) | 3.56 (4.44) |
| *Note.* M = mean, SD = standard deviation. All coaching users (*n* = 266) used coaching as their primary modality of care. | | | |
